# Amyand's Hernia: A Serendipitous Diagnosis

**DOI:** 10.1155/2013/125095

**Published:** 2013-04-04

**Authors:** S. Mewa Kinoo, M. R. Aboobakar, B. Singh

**Affiliations:** Department of Surgery, Nelson R Mandela School of Medicine, University of KwaZulu-Natal, 719 Umbilo Road, Congella 4013, Durban, South Africa

## Abstract

An Amyand's hernia refers to the presence of an appendix within an inguinal hernia sac. This uncommon finding occurs in less than 1% of all right side inguinal hernias; to date, this finding has been reported in only 14 patients with left side inguinal hernias. The preoperative diagnosis of this condition is uncommon. We report the 15th case of a left side Amyand's hernia that was diagnosed preoperatively on a contrast enema study as well as the relatively more common right-sided Amyand's hernia diagnosed serendipitously at surgery.

## 1. Introduction

The first description of an appendix in an inguinal hernia is attributed to Amyand (sergeant surgeon to King George I and II) who, in 1735, found a perforated appendix in an 11-year-old boy who presented with a right inguinal hernia and faecal fistula [1]. This was also one of the first documented descriptions of an appendectomy being performed [2]. Four years prior to this, French surgeon René Jacques Croissent de Garengeot described the presence of an appendix within the femoral hernia sac, the so-called de Garengeot hernia [2].

## 2. Case Presentations

**Case 1**

**Figure 1:**
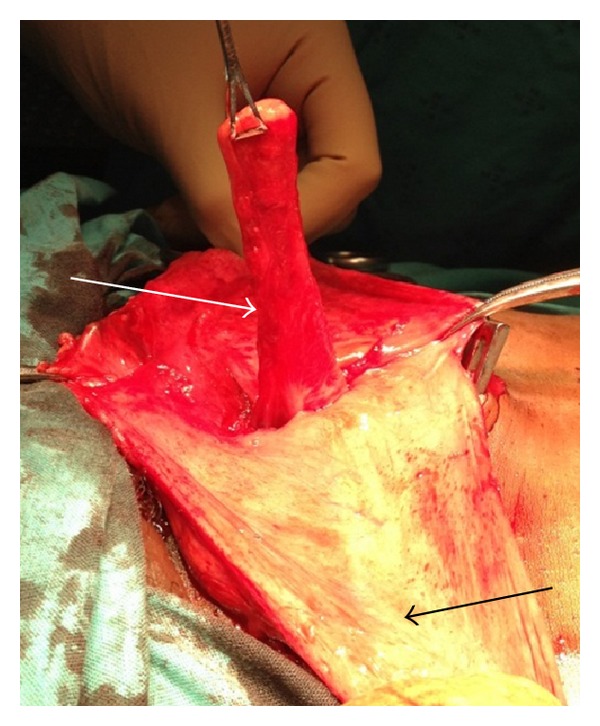
Lateral view of appendix (white arrow) within right inguinal hernia sac (indicated by black arrow).

**Figure 2:**
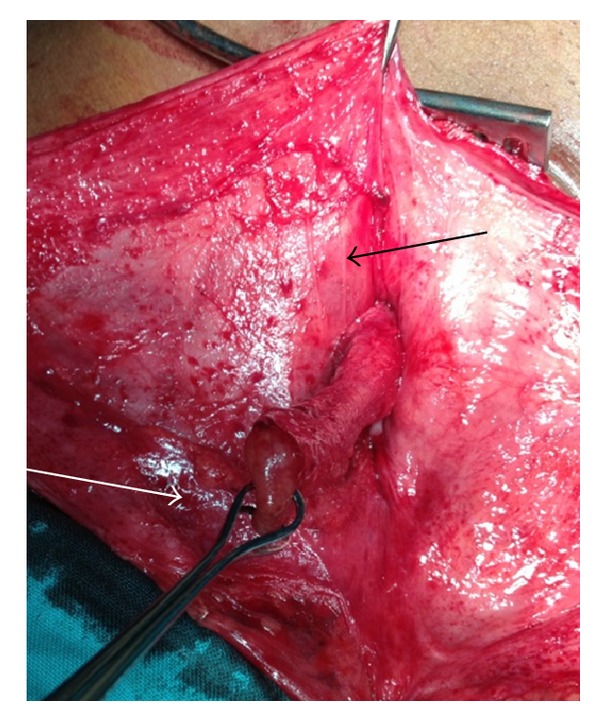
Superior view of appendix (white arrow) within right inguinal hernia sac (indicated by black arrow).

Our first case was a 44-year-old male who presented with a 6-month history of an intermittent right-sided groin swelling with intermittent mild pain and discomfort. On examination, he had a positive right groin cough impulse that reduced spontaneously and disappeared with occlusion of the internal ring. He was diagnosed with an indirect right sided inguinal hernia. At open inguinal herniorrhaphy a noninflamed appendix was noted adherent to the indirect inguinal hernia sac (see Figures 1 and 2). The appendix was freed from the sac and reduced into the abdomen. The sac was ligated and excised, and a tissue repair was undertaken. The patient had an uneventful postoperative recovery and was discharged on day 2.

**Case 2**

A 60-year-old male patient presented to his local clinic with a one-month history of an intermittent left groin swelling associated with an incarcerated inguinal hernia. The patient did not have signs and symptoms of intestinal obstruction or bowel strangulation. The hernia was reduced and the patient referred for elective hernia repair.

**Figure 3:**
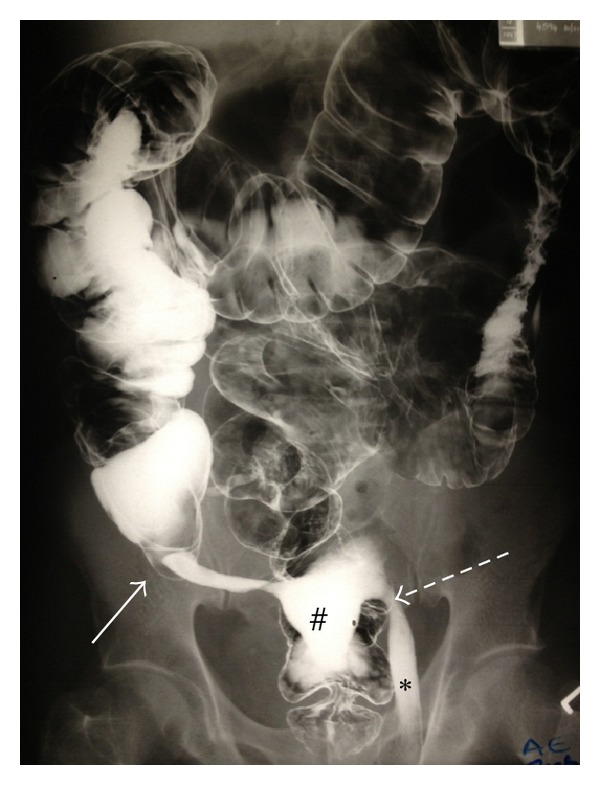
Contrast enema outlining base of appendix (white arrow) and tip of appendix at left inguinal ring (dashed arrow). Note the extravasation of contrast in pelvis (indicated by #) and in the left inguinal canal (indicated by ∗).

**Figure 4:**
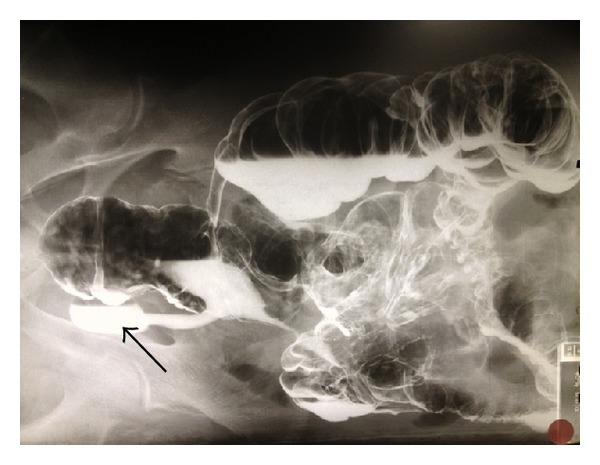
Contrast within left inguinal sac noted on decubitus positioning of the patient (indicated by black arrow).

Clinical evaluation prior to elective left inguinal herniorrhaphy revealed an easily reducible inguinal hernia. In response to the patient admitting to experiencing chronic constipation, a water-soluble contrast enema was performed. The enema demonstrated the opacification of the appendix lying adjacent to the left internal ring with extravasation of contrast into the pelvis and left inguinal canal (Figures 3 and 4).

Due to the contrast enema findings, the patient was taken for an emergency exploratory laparotomy. The appendix was noted within the sac of the left inguinal hernia with a perforation at the level of the internal ring surrounded with omentum, forming a contained pelvic abscess. The abscess was drained, an appendectomy performed, and a tissue repair of the left inguinal hernia undertaken. Histology confirmed appendicitis in a long appendix and mixed acute and chronic inflammation involving the hernia sac. The patient had an uneventful recovery and was discharged on day 7 after laparotomy.

## 3. Discussion

Right-sided Amyand's hernias occur more often than left due to the anatomical location of the appendix on the right. Left-sided Amyand's hernias are very rare; a literature review revealed only 15 reported cases of left-sided Amyand's hernias to date, including our case [3, 5–16]. Reasons postulated for the presence of left-sided Amyand's hernias may be situs inversus, malrotation, a mobile caecum, and an excessively long appendix [6]. In the case of Amyand's hernia, the appendix may be normal, inflamed, or perforated within an inguinal hernia. 

The incidence of having a normal appendix within an inguinal hernia sac is about 1%, whereas appendicitis present in an inguinal hernia is reported to be 0.1% [5]. The decision whether to perform an appendectomy has been addressed by the classification proposed by Losanoff and Basson; in the type 1 Amyand's hernias (appendix not inflamed), appendicectomy is not routinely undertaken unless the patient is young. In types 2, 3, and 4 Amyand's hernias (inflamed appendix) appendicectomy is routine (see Table 1 [3, 4]). 

Johari et al. suggested that appendectomies for left-sided hernias be routinely performed irrespective if inflamed or not, as a future appendicitis may pose a diagnostic doubt due to the position of the appendix. The use of a prosthetic mesh is contraindicated in inflammation or infection. 

While CT scan may prompt a preoperative diagnosis as in those presenting with an acute abdomen presentations due to incarcerated or strangulated hernias, the diagnosis of Amyand hernia is usually made intraoperatively [17].

## 4. Conclusion

Although Amyand's hernias are a rare occurrence, the attendant surgeon should be vigilant about its presence as it may be mistaken for an acute hydrocele, testicular torsion, or epididymo-orchitis. Preoperative diagnosis is not common because imaging is not routine and is usually performed to exclude conditions that predispose to inguinal hernia formation. Left-sided hernias may require postoperative imaging to exclude situs inversus or a gut malrotation. The decision to perform an appendectomy and type of repair depend on the clinical scenario and are guided by Losanoff and Basson's criteria.

## Figures and Tables

**Table 1:** Classification of Amyand's hernias after Losanoff and Basson [3, 4].

Classification	Description	Surgical management
Type 1	Normal appendix within an inguinal hernia	Hernia reduction, mesh repair, appendectomy in young patients

Type 2	Acute appendicitis within an inguinal hernia, no abdominal sepsis	Appendectomy through hernia, primary endogenous repair of hernia, no mesh

Type 3	Acute appendicitis within an inguinal hernia, abdominal wall or peritoneal sepsis	Laparotomy, appendectomy, primary repair of hernia, no mesh

Type 4	Acute appendicitis within an inguinal hernia, related or unrelated abdominal pathology	Manage as types 1 to 3 hernia, investigate or treat second pathology as appropriate
